# The effect of somatosensory alpha transcranial alternating current stimulation on pain empathy is trait empathy and gender dependent

**DOI:** 10.1111/cns.13631

**Published:** 2021-03-19

**Authors:** Peipei Wang, Minjia Zhu, Shaohua Mo, Xiaoli Li, Jing Wang

**Affiliations:** ^1^ Department of Neurobiology, School of Basic Medical Sciences Capital Medical University Beijing China; ^2^ Department of Orthodontics, School of Stomatology Capital Medical University Beijing China; ^3^ Department of Neurosurgery Beijing Tiantan Hospital, Capital Medical University Beijing China; ^4^ State Key Laboratory of Cognitive Neuroscience and Learning Beijing Normal University Beijing China

**Keywords:** Alpha ERD, empathy concern, pain empathy, transcranial alternating current stimulation

## Abstract

**Background:**

Pain empathy enables a person to experience and understand other's pain state by observing others in pain condition. Such prosocial ability is deficient in many psychopathological disorders. Somatosensory alpha suppression is considered as neural correlates of pain empathy and is hypothesized as a target for enhancement of pain empathy. Researches demonstrated that alpha suppression could be enhanced by transcranial alternating current stimulation (tACS) at alpha frequency non‐invasively.

**Aims:**

We applied alpha tACS over the primary somatosensory cortex of healthy subjects to investigate whether alpha tACS is able to enhance the pain empathy performance.

**Results:**

The results showed that there was no difference of pain empathy performance between alpha tACS and sham tACS either when tACS was applied during the task or before task. While in the alpha tACS group, the pain empathy performance was positively correlated with empathic concern of male subjects, the sub‐component of personal trait empathy.

**Conclusions:**

Alpha tACS cannot alter the empathy performance overall, but the modulation effect of alpha tACS on pain empathy is dependent on the gender and trait empathy of subjects.

## Graphical abstract

We tested whether alpha tACS could enhance the empathy ability, which is deficit in many psychopathological disorders such as autism and alexithymia. We did not find overall modulation effect of alpha tACS on pain empathy performance either online or offline. While the pain empathy was positively correlated with empathy concern of male subjects indicating the gender and trait empathy‐dependent characteristics of alpha tACS on pain empathy.

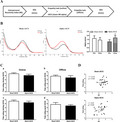

## 1 INTRODUCTION

Empathy is an ability to recognize and share others feelings, which is core for successfully daily social interaction.^1^ The empathy for pain refers to understanding or recognizing pain perception of others by observing others experiencing pain,^2^, ^3^ and it was found that the empathy for pain is particular important for human being's social activity. For example, empathizing with pain of another person will trigger prosocial actions.^2^ On the other hand, the empathy‐related deficits are associated with psychopathological disorders, such as autism spectrum disorder and schizophrenia.^4^, ^5^ It is of interest to develop a reliable method to increase empathy ability to promote human being's prosocial actions.

The underlying neural mechanism of empathy for pain has been widely studied. Accumulating evidence indicated that response to pain in others not only revealed in emotional pain pathway but also in sensory pain matrix of observers. Multiple functional MRI studies found that the primary somatosensory cortex (S1) is activated when somebody is watching others in painful conditions,^6^, ^7^, ^8^, ^9^ and the prosocial behaviors are inferenced when the activity of S1 is suppressed by TMS.^10^ Neuroscientists further found oscillatory activity at alpha frequency band (~10 Hz) over the S1 participates in the processing of empathy for pain.^11^, ^12^ When subjects are observing painful pictures of others, electroencephalographic (EEG) exhibits decreased alpha power compared with baseline and non‐painful conditions over S1, that is, alpha event‐related desynchronization (ERD).^12^, ^13^, ^14^, ^15^, ^16^ The alpha ERD in the somatosensory positively correlates with empathy performance, that is, pain ratings from others’ perceptive, indicating the correlational relationship between empathy for pain and the suppression of the somatosensory alpha activity. These results indicate that the alpha ERD in the somatosensory cortex is associated with pain empathy.

Transcranial electric stimulation is a non‐invasive technique to modulate the brain activity. Our previous research demonstrated that transcranial direct current stimulation could improve the empathy ability,^17^ while the direct current stimulation cannot modulate the frequency‐specific activity to produce the mechanism‐based modulation effect. Transcranial alternating current stimulation (tACS) applies alternating currents at specific frequency to the scalp.^18^ In particularly, tACS could manipulate the brain oscillatory activity in a frequency‐specific manner.^19^, ^20^ Previous studies showed that tACS with alpha frequency could enhance alpha power of EEG^21^, ^22^ and change pain perceptions.^23^ Importantly, recent researches demonstrate continuous alpha tACS at resting state increase ongoing alpha power and alpha ERD.^24^, ^25^ These results suggest that applying alpha tACS at resting state could be taken as manipulators of alpha ERD to improve the empathy ability. This study is to test whether alpha tACS over the S1 can enhance the ability of empathy for pain.

## 2 MATERIAL AND METHODS

### 2.1 Subjects

Fifty‐two healthy subjects were recruited, aged 18 to 30 years (21.5 ± 3.39, 27 females and 25 males). Subjects had right‐handedness, normal vision, or corrected vision, and they were self‐reported to have no history of neurological disease and mental disorders. There were no metal implantations in their bodies as well. Subjects signed an informed consent form before the experiment in accordance with the ethical principles of the Declaration of Helsinki. The consent form was approved by the Ethics Committee of Capital Medical University.

### 2.2 Trait empathy: Interpersonal Reactivity Index.

Interpersonal Reactivity Index‐Chinese (IRI‐C) was used measure trait empathy. This scale was revised by Siu AMH^26^ based on the Interpersonal Reactivity Index edited by.^27^ There are 22 items in total. A Likert 5‐point scale is used, each scored from 0 (completely inconsistent) to 4 (fully met), and the reverse question is scored in the opposite direction. It was used to evaluate multiple dimensions of empathy including perspective‐taking (PT), fantasy scale (FS), empathic concern (EC), and personal distress (PD). The subjects gave their scores for each item (Table 1).

**TABLE 1:** Interpersonal reactivity index scores

Group	FS	EC	PT	PD	Total
Anodal	2.10 ± 0.69	2.27 ± 0.57	2.41 ± 0.59	2.10 ± 0.76	8.88 ± 0.66
Sham	2.07 ± 0.69	2.64 ± 0.38**	2.51 ± 0.53	2.20 ± 0.49	9.42 ± 0.58

Abbreviations: EC, empathic concern; FS, fantasy scale; PD, personal distress; PT, perspective taking.

** *p* < 0.01.

### 2.3 Empathy task

The task programmed using the E‐prime software version 2 (Psychology Software Tools, Inc.). Subjects were presented with pictures either depicting right hands being injured or being intact. Painful and non‐painful pictures were selected from pictures that were validated in previously published studies.^17^, ^28^ Each picture lasted for 1 s and 20 pictures appeared randomly. How painful the person felt after the presentation of each picture. The subjects were instructed to evaluate the pain intensity of the person in the picture on a scale of 0–9 (0: no pain at all, 9: extreme pain).

### 2.4 EEG recording and processing

64‐channel EEG were recorded (BrainAmp MR32, Brain Products GmbH). The lead method used the international 10–20 standard system. The Cz was set as the online reference electrode. The impedance of each channel was kept <5 kΩ. The sampling frequency was 1000 Hz. The data were preprocessed using EEGLAB (The Mathworks; http://sccn.ucsd.edu/eeglab/). The band‐pass filter was set between 1 and 70 and the 50 Hz noise was notch filtered. Bad channels were manually removed and interpolated. Data were then referenced to the average reference. Power spectrum was estimated using the multitaper method.^29^ Data were segmented into epochs of 2 s with a sliding window of 1 s. Slepian tapers were used to obtain a frequency smoothing of 2 Hz, and the power was averaged over segments and tapers. According to previous study,^14^ theta (2–7 Hz), alpha (8–13 Hz) and beta (13–20 Hz) power were averaged over channels (C3,CP3,C1,CP1,CPz) as the primary somatosensory cortex.

### 2.5 tACS

Alternating Current was employed by a battery‐driven, alternating current simulator (Jianxi Huaheng Jingxing Medical Technology Co. Ltd.) with two rubber electrodes (50 × 70 mm). Because a previous finding reported that alpha ERD was increased by 1 mA tACS for 5 mins,^30^ we applied 1 mA alternating current (peak‐to‐peak amplitude, sinusoidal waveform) at 10 Hz for 10 min as the alpha tACS stimulation; an anodal electrode was placed over the somatosensory cortex, the 2 cm posterior to C3 according to the international 10–20 system. An cathodal electrode was placed on the upper orbital gyrus. During the stimulation, the impedance was kept below 10 kΩ between skin and electrode. The setting of sham tACS stimulation was the same to the alpha tACS stimulation but with 1 mA current for 30 s to mimic transient tingling sensations associated with the onset of active stimulation for the purpose of blindness.^31^

### 2.6 Procedure

**FIGURE 1:**
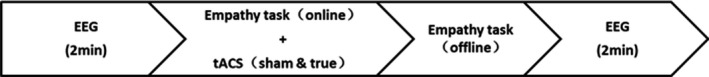
The diagram of the experiment procedures

After completing the Interpersonal Reactivity Index (IRI), the subjects were randomly arranged into two experimental groups: the alpha tACS stimulation group and the sham stimulation group. The subjects were blind to the grouping. Then, 2 min of EEG were recorded and followed by tACS stimulation. At the last 2 min, the subjects performed the empathy task (on‐line task). After the task and stimulation, the empathy task and EEG recording were conducted again (off‐line task). The resting‐state EEG was recorded for 19 subjects before and after tasks (Figure 1).

### 2.7 Statistical analysis

The Shapiro‐Wilk was applied for testing the normality of all data. Since all variables met the normal distribution and sphericity, two‐way repeated ANOVA with recording time as a repeated factor was used to estimate the power changes. Student *t*‐test was used to compare the difference of pain ratings between the two groups. Pearson correlation and linear regression were used to estimate the relationship between empathic concern and pain ratings. *p* < 0.05 was considered as statistically significant.

## 3 RESULTS

**FIGURE 2:**
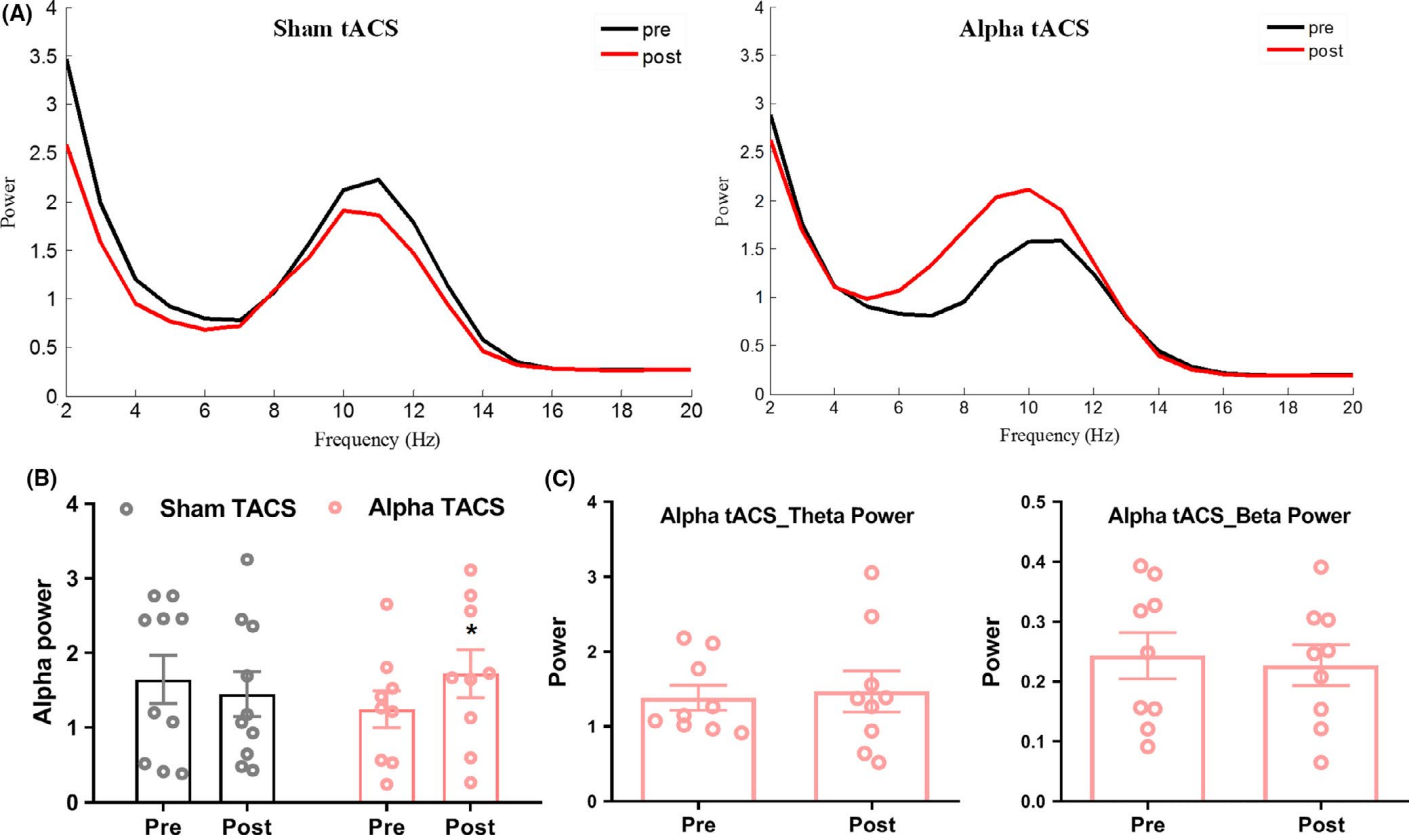
Power spectrum at alpha frequency increased after alpha tACS. A: Power spectrum before and after sham tACS and alpha tACS. B: Changes of power at alpha frequency band between groups. C: Changes of theta and beta frequency band between groups* *p* < 0.05[Colour figure can be viewed at wileyonlinelibrary.com]

We calculated the power spectrum over the S1 from 1 to 70 Hz before and after stimulation for both the sham tACS and alpha tACS group (Figure 2A). Compared with the sham tACS group, alpha power of S1 increased in the alpha tACS group (Figure 2B). Two‐way repeated ANOVA found a significant interaction (*F*_(1,17_) = 5.0, *p* < 0.05), while there was no significantly changes for theta and beta power between two groups (*F*_(1,17)_ = 1.67; *F*_(1,17)_ = 0.05, *p* > 0.05, Figure 2C). These results demonstrated that alpha tACS increases the ongoing alpha power.

**FIGURE 3:**
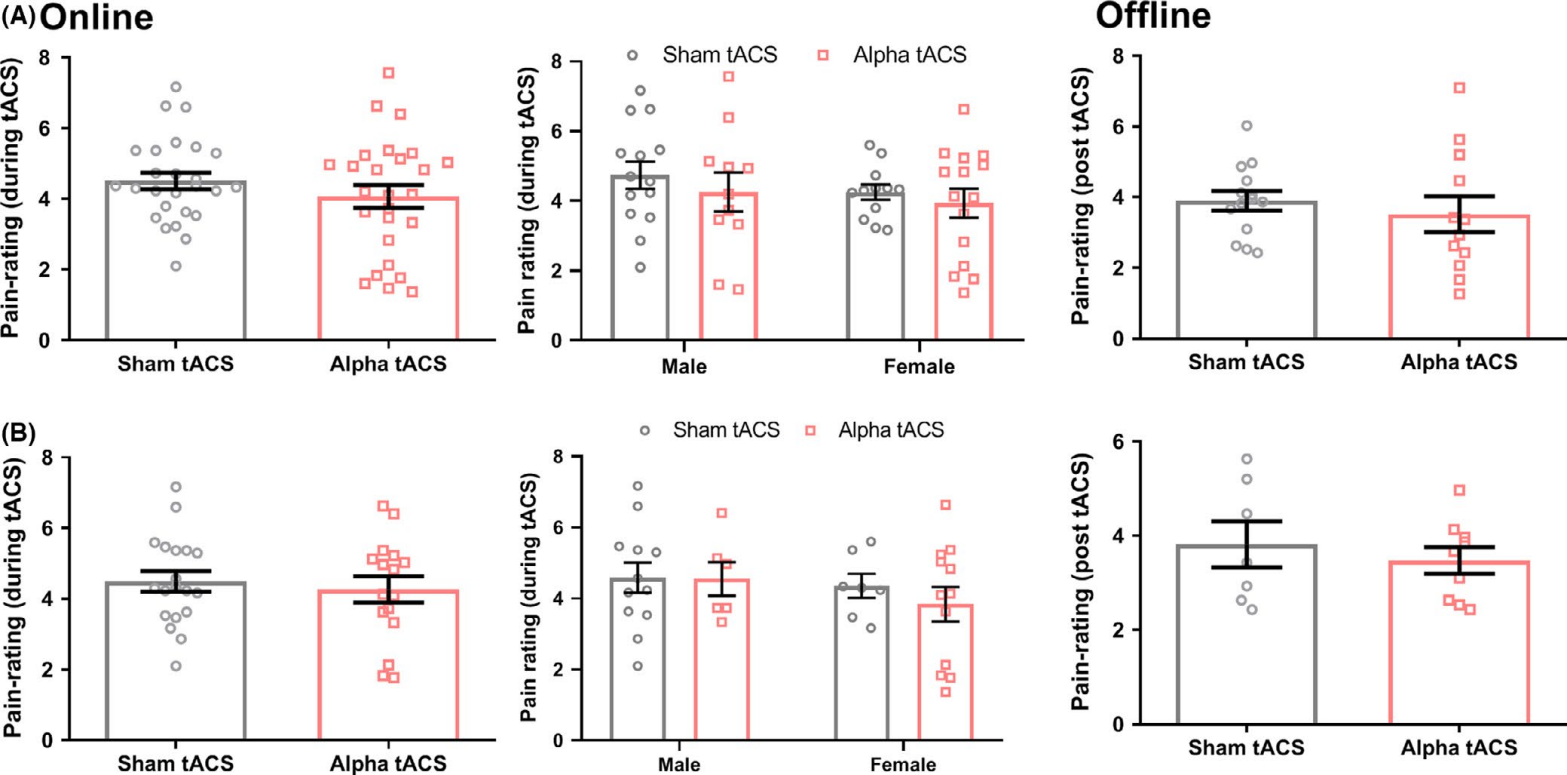
Pain empathy performance unchanged in the online and offline task. A: Pain ratings in the online task and offline task in both the sham tACS and alpha tACS group. B: The EC corrected pain ratings were shown for the online task and the offline task [Colour figure can be viewed at wileyonlinelibrary.com]

In order to investigate the online effect of alpha tACS on pain empathy, we compared the ratings of painful picture for other's pain during the stimulation between the sham tACS group and alpha tACS group. As shown in Figure 3A, pain ratings between the alpha tACS and sham tACS were not significantly different in the online task (*t*_(50)_ = 1.09, *p* = 0.28). Similarly, no difference found in the offline task (*t*_(24)_ = 0.68, *p* = 0.50). As the difference of the empathy ability is frequently reported,^32^, ^33^ we compared gender differences in pain empathy. The online task did not show significant difference between alpha and sham tACS in the male and female subjects (*F*_(1,48)_ = 0.04, *p* = 0.84) (Figure 3A). Because the empathic concern (EC) of IRI was not balanced between two groups (*t*_(50)_ = 2.75, *p* < 0.01, Table 1), we selected pain ratings from those with balanced EC values (between 2.0 and 3.0) for analysis in order to rule out this confounding factor. The corrected results still did not showed significant difference of pain ratings in either the online or the offline task between groups (*t*_(16)_ = 0.73, *p* = 0.47; *t*_(14)_ = 0.64, *p* = 0.53). No significant difference was found in male and female subjects (*F*_(1,33)_ = 0.24, *p* = 0.63) (Figure 3B).

**FIGURE 4:**
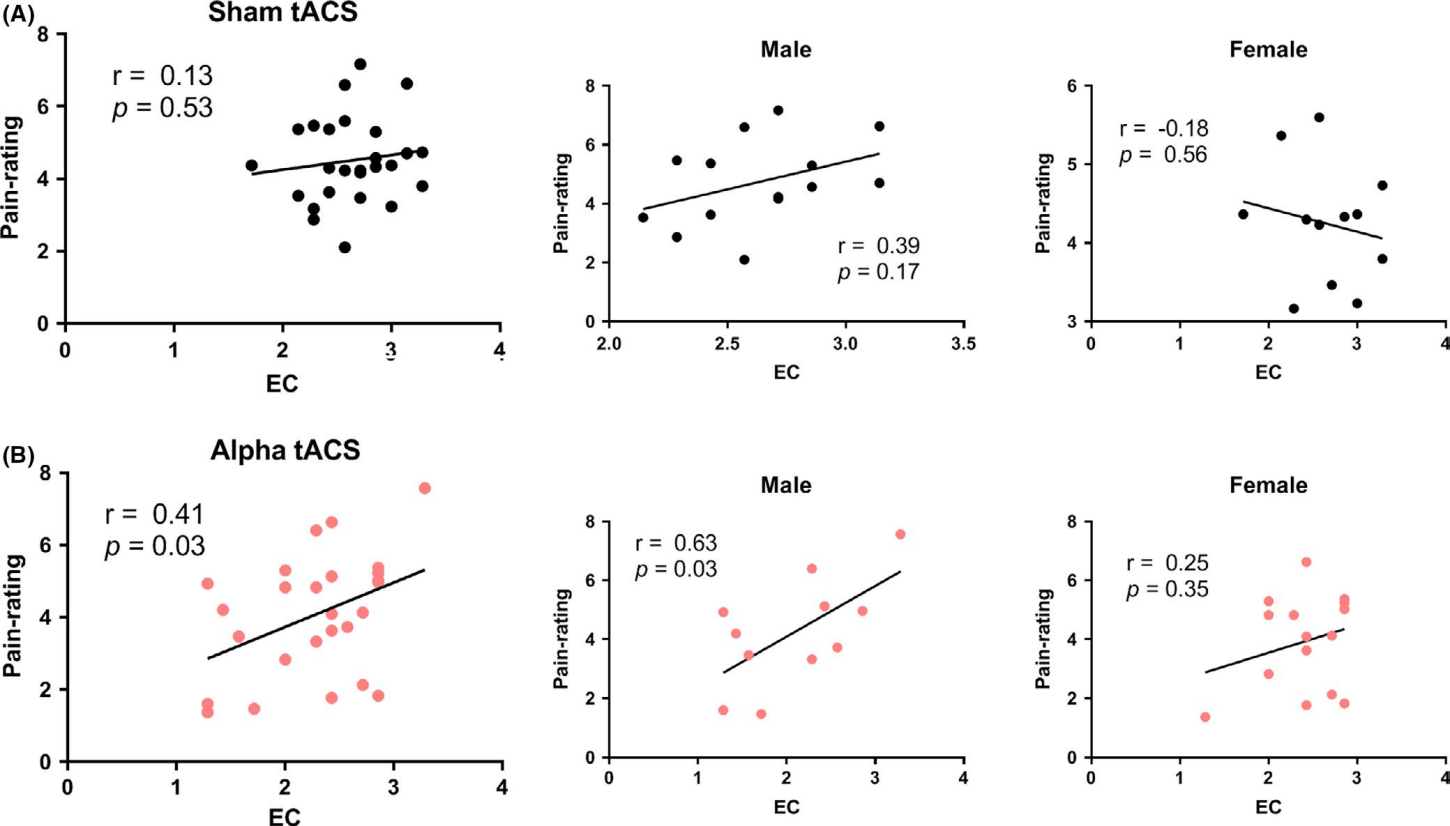
Correlation between EC and pain empathy performance. Pain ratings for other's pain were positively correlated with EC in the alpha tACS group in male subjects (B) but not in the sham tACS group (A) [Colour figure can be viewed at wileyonlinelibrary.com]

Previous researches demonstrate alpha ERD in empathy task is associated with trait empathy of subjects in particular the empathic concern.^34^, ^35^ So, we speculated whether the modulation effect of alpha tACS relates to personal trait empathy. And we analyzed the empathy behavior of two groups in terms of subject's empathic concern. As shown in Figure 4A, it was found that pain ratings positively correlate with EC in the alpha tACS (*r* = 0.41, *p* = 0.03), indicating greater empathy for pain in subjects with higher empathic concern under alpha tACS. Further linear regression found EC predicted pain rating (*r* ^2^ = 0.17, *p* < 0.05). When we correlated the EC and tACS for male and female separately, such positive correlation just showed in male group (*r* = 0.63, *p* = 0.03) but not in female group (*r* = 0.25, *p* = 0.35). While, this phenomenon did not show in the sham tACS group (*r* = 0.13, *p* = 0.53) for both male subjects and female subjects (Figure 4B). It indicated the modulation effect of tACS on pain empathy varies with empathic concern and gender of subjects.

## 4 DISCUSSION

This study found that the modulation effect of tACS at alpha frequency on empathy for pain was dependent on the subject's empathic concern and sex, though the difference was not found overall.

### 4.1 Alpha ERD in the somatosensory cortex is not the necessary mechanism for pain empathy

Reports have shown that alpha ERD in the somatosensory cortex associated with empathy tasks. Because alpha tACS can enhance alpha ERD, we tested whether alpha tACS improve empathic performance in the current study. Unexpectedly, results did not show that alpha tACS enhanced pain empathy. Such negative results may have two possible explanations. One possible reason is that alpha tACS did not increase alpha ERD in the empathy task. In the current study, we recorded and analyzed ongoing alpha activity before and after alpha tACS. Results showed that power increased exclusively at alpha frequency band in the somatosensory cortex after alpha tACS, which is in line with previous reports.^21^, ^22^, ^24^, ^25^ While due to the strong artifact during the tACS stimulation, we did not record the event‐related activity and analyze the alpha ERD, that is, there was no direct evidence of enhanced alpha ERD by alpha tACS. However, based on previous evidences that the enhanced ongoing alpha power that is induced by tACS is always accompanied with enhanced alpha ERD,^24^, ^25^ it is highly possible that alpha tACS increased alpha ERD in this research. Another possible reason is that alpha ERD in the somatosensory cortex is not the necessary mechanism for pain empathy. Although alpha ERD in the somatosensory cortex was thought to participate in pain empathy, no causal evidence supported this hypothesis. Our results showed that the empathy performance did not change when alpha ERD was manipulated in the somatosensory cortex, suggesting it is not necessary for pain empathy. This speculation is consistent with recent findings and reviews, which emphasize that alpha ERD in the somatosensory cortex is not an strong index for the mirror neuron system that underlies the empathy processing.^36^, ^37^ Besides, the age of subjects in our study (averaged 21.5 years) is close to the age of subjects in a study which showed younger subjects (averaged 17 years) did not employ alpha ERD in the somatosensory cortex in pain empathy task. Accordingly, alpha ERD in the somatosensory cortex is probably not necessary neural correlates of empathy at least in subjects with younger age.

### 4.2 The effect of tACS is dependent on trait empathy and sex

It is interesting to find the pain empathy in the alpha tACS group was positively correlated with empathic concern. Empathic concern is a sub‐component of IRI, the widely used measurement of trait empathy. These findings infer that the modulation effect of somatosensory alpha tACS is dependent on trait empathy of subjects, stronger empathic concern with higher pain empathy. More interestingly, such correlation exhibited just in men but not in women, indicating the trait empathy and gender‐dependent characteristics of tACS. Such state‐dependent characteristics of tACS also accords with other findings which showed that the brain activity has critical impact on the efficacy of tACS.^21^ Based on these findings, we hypothesize that the gender difference of tACS related to gender difference in empathic neural processing. Indeed, EEG and fMRI study reported that compared to male subjects, female subjects have stronger alpha ERD and stronger activation of the inferior frontal cortex in.^38^, ^39^, ^40^ It deserves further study to explore why male subjects show trait empathy‐dependent characteristics of tACS. Thus, measuring the IRI is of importance for effective usage in patients with empathy deficient. In addition, our findings suggested that alpha ERD in the somatosensory cortex may not serve the underlying mechanism of sub‐groups.

### 4.3 tACS at resting state do not modulate pain empathy

In the offline take, we applied alpha tACS at resting state. The result showed that pain empathy did not changed, indicating that resting alpha activity did not participant in the empathy behavior. A previous research investigated whether resting alpha activity is responsible for empathy. The result showed that resting alpha activity is not related to the mirror neuron system which underlies empathy processing.^36^ Our result is consistent with these findings and supported it from the modulation perspective.

### 4.4 The different effect of alpha tACS and transcranial direct current stimulation (TDCS) on empathy

Transcranial direct current stimulation is another important form of transcranial current stimulation. Recent researches indicate that transcranial direct current stimulation (tDCS) improved empathy performance in healthy subjects^17^ and shows effectiveness in psychological disorders such as depression which is associated with empathy deficit.^41^, ^42^ Such differential findings with our results suggest the effect of tDCS and tACS on empathy, and its related disorders is different. It deserves further investigation to test whether alpha tACS is effective in empathy‐related psychological disorders, especially with personalized tACS stimulation.^43^

## 5 LIMITATIONS

Several limitations should take into consideration. Although tACS at 10 Hz increased alpha power, 10 Hz stimulation may do not target individually meaningful alpha activity since alpha frequency varies individually. In addition, due to the large artifact induced by tACS during the EEG recording, the EEG before and after task is recorded in most studies. The lack of direct evidence from event‐related alpha suppression in the current study should be take caution, though recent MEG findings showed tACS increased both alpha power and event‐related alpha suppression simultaneously.

In conclusion, our results found that somatosensory alpha tACS does not change pain empathy in overall but show gender and trait empathy‐dependent modulation effect. Our results suggest the potential application of alpha tACS in empathy disorders and deepen the understanding the role of alpha oscillatory brain activity in empathy for pain.

## DISCLOSURE

We declared that the research was conducted in the absence of any commercial or financial relationships that could be construed as a potential conflict of interest. There are no competing interests exists.

## AUTHOR CONTRIBUTIONS

M‐JZ, P‐PW, and S‐HM performed the experiments. M‐JZ, P‐PW, and JW analyzed the data. M‐JZ, P‐PW, and JW prepared the manuscript. X‐LL and JW designed the study.

## Data Availability

The data that support the findings of this study are available on request from the corresponding author. The data are not publicly available due to privacy or ethical restrictions.
